# Conductive Nerve Conduits With Orientated Topological Structures From Ice‐Templating Technology

**DOI:** 10.1002/smmd.70012

**Published:** 2025-06-30

**Authors:** Hui Zhang, Kaichen Wang, Dongyu Xu, Shuangshuang Miao, Yanhong Dai, Panmiao Liu, Huan Wang

**Affiliations:** ^1^ Department of Otolaryngology Head and Neck Surgery Nanjing Drum Tower Hospital School of Biological Science and Medical Engineering Southeast University Nanjing China; ^2^ Department of Anesthesiology Pain and Perioperative Medicine The First Affiliated Hospital of Zhengzhou University Zhengzhou China; ^3^ The Eighth Affiliated Hospital Sun Yat‐sen University Shenzhen China

**Keywords:** conductivity, ice‐templating, nerve guidance conduits, nerve regeneration, topological structure

## Abstract

Artificial nerve conduits hold significant promise for treating nerve injuries, with researchers focusing on simplifying techniques to harness microstructures and functions to improve their therapeutic outcomes. Here, a type of conductive nerve guidance conduit (NGC) with orientated topological structures from ice‐templating technology is presented for promoting peripheral nerve regeneration. Based on a temperature gradient generated by a thermoelectric cooling platform, conductive carbon nanotubes (CNTs) and methacrylated gelatin are introduced into the ice crystal template to create conductive conduits with unique oriented structures. Ascribed to such structures, together with the great conductivity of CNTs and the loaded nerve growth factors, the obtained conduits can direct the neurite extension and facilitate the differentiation and growth of nerve cells. By constructing rat models with long‐segment sciatic nerve defects, it was confirmed that such conductive NGCs can effectively improve injured nerve regeneration and motor function recovery. These features reveal the practical application value and broad prospect of our prepared NGCs in improving peripheral nerve regeneration.

## Summary

- The orientated topological structures from the ice‐templating technology promote directional neurite extension.
- The incorporation of carbon nanotubes provides electrical conductivity, while nerve growth factors further enhance nerve regenerative capabilities.
- The NGCs significantly improve regeneration and functional recovery in sciatic nerve defects.

## Graphical abstract

**Conductive nerve conduits featuring oriented topological structures** are engineered through ice‐templating technology, utilizing hydrogels and temperature gradients. Such conduits enhance peripheral nerve regeneration by integrating conductivity, aligned architecture, and growth factors, as demonstrated by improved motor recovery in rat models with sciatic nerve defects, indicating their clinical potential for treating nerve injuries.

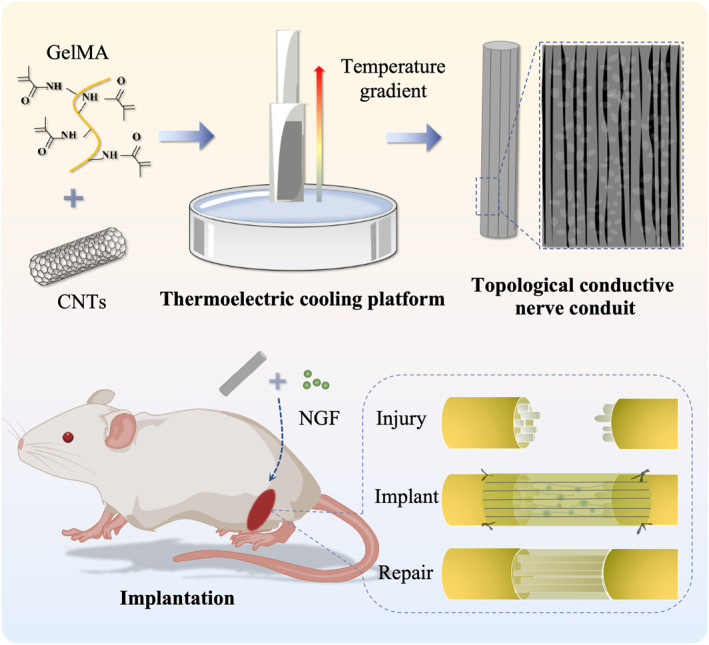

## 1 Introduction

Peripheral nerve injury (PNI) is a highly prevalent condition globally, imposing significant medical and economic burdens on society [1]. Serious PNI frequently results in the disruption of the connection between the central nervous system and various surrounding tissue, thereby impeding information transmission during body movements [2]. Recently, multivarious therapeutic strategies have been proposed for PNI treatment, among which the use of bioactive molecules such as nerve growth factors (NGF) and other neurotrophins has been confirmed to positively affect nerve regeneration [3]. Despite some progress, medication alone is often insufficient for restoring injured nerves with long‐segment defects. In contrast, applying nerve guidance conduits (NGCs) derived from various biomaterials represents an effective avenue for promoting defective nerve regeneration [4]. Especially, NGCs compounding therapeutic agents have been exploited to promote the differentiation and migration of neurons at the damaged site, thus enhancing nerve regeneration outcomes [5]. However, many existing NGCs lack precise micro‐/nano‐structure designs for directing neurite elongation and ignore the supply of electrical environment required for optimal neuron outgrowth, resulting in unsatisfactory regeneration outcomes and functional recovery. Thus, novel NGCs with topographic cues and conductive properties are still greatly anticipated.

**FIGURE 1:**
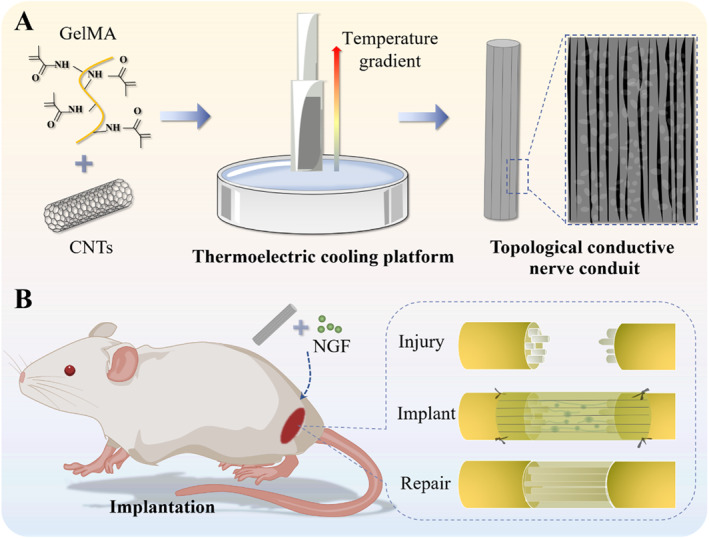
Schematic illustrations of the preparation (A) and application (B) of conductive NGCs via ice‐templating technology.

In this paper, we proposed a type of conductive nerve conduit with orientated topological structures from ice‐templating technology for PNI repair, as schemed in Figure 1. Under the temperature gradient generated by the Peltier thermoelectric cooling platform, directional growth of ice crystals in the precursor solution can be achieved, thereby generating a unidirectional structure [6]. Based on such a technique, carbon nanotubes (CNTs) and methacrylated gelatin (GelMA) were utilized to prepare topologically conductive conduits, which could induce oriented neuron growth within restricted regions. Furthermore, the excellent conductivity of CNTs enabled the conduit to provide electrical signals for neuron growth, thus promoting the regeneration of injured nerves [7]. In particular, NGF was loaded into the pores derived from ice‐templates and its stable release from conduits was confirmed. It was also verified that such conductive NGCs facilitated cell differentiation and directional neurite extension. Further animal experiments demonstrated that the prepared NGCs effectively promoted nerve repair and functional recovery in rat models with long‐segment nerve defects. These results indicated the important clinical value of the proposed NGCs in promoting nerve regeneration.

## 2 Results and Disccussion

**FIGURE 2:**
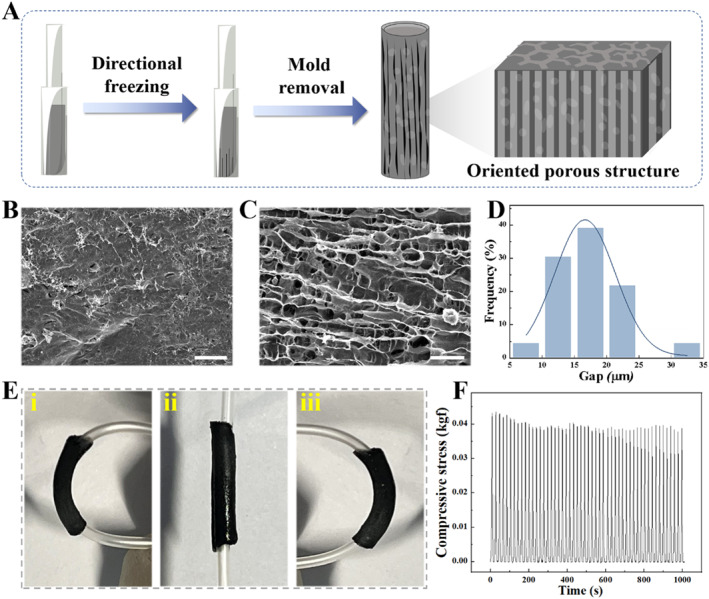
(A) Illustration of the preparation process for conductive NGC featuring topological structures using ice‐templating technology. (B) SEM image of the interior surface of normal NGC without directional structure. The scale bar is 100 μm. (C) SEM image of the interior surface of NGC with directional structure. The scale bar is 100 μm. (D) Distribution of gap size of NGC with directional structure. (E) Pictures of the NGC undergoing bending. (F) Durability test of the prepared NGC, involving 50 cycles of compression at a 40% strain.

In a typical experiment, the conductive NGCs with orientated topological structures were fabricated by using ice‐templating technology. In detail, a sandwich catheter perfusion mold composed of two glass capillaries with different diameters was constructed and placed on a Peltier thermoelectric cooling platform. In order to avoid the temperature varying between the cooling platform and the surrounding environment, a sponge device with thermal insulation was placed outside the double‐layer glass capillary mold. Then, a precursor solution composed of GelMA with low molecular weight peptides (2000 Da), and CNTs was injected into the above catheter perfusion mold. Since the temperature was transferred from the Peltier platform from the bottom up, the ice crystals grow into a regular longitudinal arrangement in the pregel. After ultraviolet (UV) light irradiation and mold removal, the NGCs with oriented topological structures were generated, as illustrated in Figure 2A. It was seen through scanning electron microscopy (SEM) that the internal wall of the resultant NGC showed an obvious directional structure based on the temperature gradient, whereas the interwall of the normal NGC without a temperature gradient had a disordered arrangement (Figure 2B,C). Besides, there were abundant porous structures within the NGC due to the space‐occupying effect of ice crystals and cross‐linked networks of GelMA, which contributed to drug loading and substance exchange (Figure S1). This result proved that the temperature gradient generated by the Peltier platform had a significant effect on the directional structure of NGCs.

Notably, we focused on exploring the influence of CNT concentration on the topological structure of the hydrogel formed by ice‐templating technology. A series of hydrogel precursor solutions containing different concentrations of CNTs were injected into the perfusion mold on a Peltier platform to form hydrogel conduits, followed by polymerization, mold removal, and freeze‐drying. It was found that the resistance values of the hydrogel conduit decreased with the increase of CNTs concentration (Figure S2). Intriguingly, at the CNT concentration larger than 40 mg/mL, the electrical resistance plateaued, exhibiting no significant variation. Besides, we assessed multiple factors affecting the gap size of the formed topological structures in hydrogel conduits. On the one hand, it was observed that the gap distance between the ridges of the topological structures could be adjusted by the concentration of CNTs. Compared with the pure GelMA conduit, the gap distance after the incorporation of CNTs became smaller (Figure S3A). On the other hand, it was demonstrated that the gap distance of the structures decreased with the decrease in thermoelectric cooling platform temperature (Figure S3B). Considering the parametric optimization balancing conductivity, mechanical strength and topological structures, the concentration of CNTs was selected at 40 mg/mL, in conjunction with the Peltier platform at −40°C, in which case the gap distance of the topological structure and electrical property were more suitable for the growth of nerve cells. Under optimal conditions, the gap size of the topology structure in the NGCs was concentrated between 15 and 25 μm, and the orientation was concentrated in a narrow angular range (Figures 2D and S3C).

To evaluate the mechanical performance of the prepared NGCs based on ice‐templating technology, compressing and bending tests were conducted. It was found that the NGCs allowed bending without incurring structural damage and fracture, as shown in Figure 2E. Additionally, the NGCs with topological structures exhibited excellent mechanical resilience, maintaining their shape and integrity under repeated compressive loading cycles, as shown in Figure 2F. These results highlight the mechanical suitability of the fabricated NGCs, which is crucial for withstanding the dynamic mechanical stresses encountered in vivo, such as those from muscle contractions or limb movements. The good durability and deformation resistance suggest that these NGCs could serve as promising alternatives to autologous nerve grafts, which could avoid mechanical deformations during organism activities, thus providing support for regenerated nerve tissues.

**FIGURE 3:**
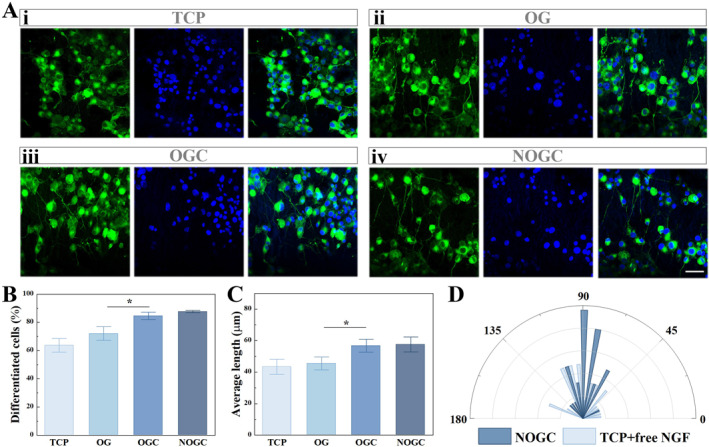
(A) Fluorescence images of PC12 cells cultured on various substrates. Scale bar is 50 μm. (B) Statistical analysis of the proportion of cell differentiation in different groups. (C) Statistics of neurites of PC‐12 cells in different groups. (D) Statistical analysis of cell neurite angle on TCP and NOGC scaffold. *p < 0.05.

Subsequently, we explored the regulatory effect of conductive hydrogels on the differentiation of pheochromocytoma 12 (PC12) cells. In detail, PC12 cells were cultured on different substrates, namely tissue culture polystyrene (TCP), oriented GelMA (OG) scaffold, oriented GelMA/CNTs (OGC) scaffold, as well as NGF‐loaded OGC (NOGC) scaffold, wherein the cells in the first three groups were treated with free NGF. After seven‐day‐cultivation, the cells were collected and immunostained, which were then observed and recorded through a laser confocal scanning microscope. It was found that PC12 cells in the TCP group exhibited a disordered distribution, while the cellular neurites elongated along the topological structures of the GelMA hydrogel substrates (Figure 3A‐i,ii). Besides, it was observed that the incorporation of CNTs into the scaffold contributed to cell differentiation on the substrates (Figure 3A‐iii). Due to the positive effect of NGF on the outgrowth and differentiation of PC12 cells, we considered loading NGF into the obtained OGC hydrogels to further enhance their performance. As a result, PC12 cells cultured on the NOGC scaffold exhibited similar neurite outgrowth to the free NGF‐added OGC scaffold, revealing the biological activity of NGF released from the substrates (Figure 3A‐iv). By measuring the differentiated cells and neurite length in different groups, it was demonstrated that the electrical conductivity of the integrated CNTs improved the differentiation of PC12 cells (Figure 3B,C). It was also seen that the distribution range of cellular neurites could be concentrated within a narrow angle range under the action of topological structures (Figure 3D). These results demonstrated the significant synergistic effect of CNTs, NGF and topological morphology on the differentiation and outgrowth of nerve cells.

**FIGURE 4:**
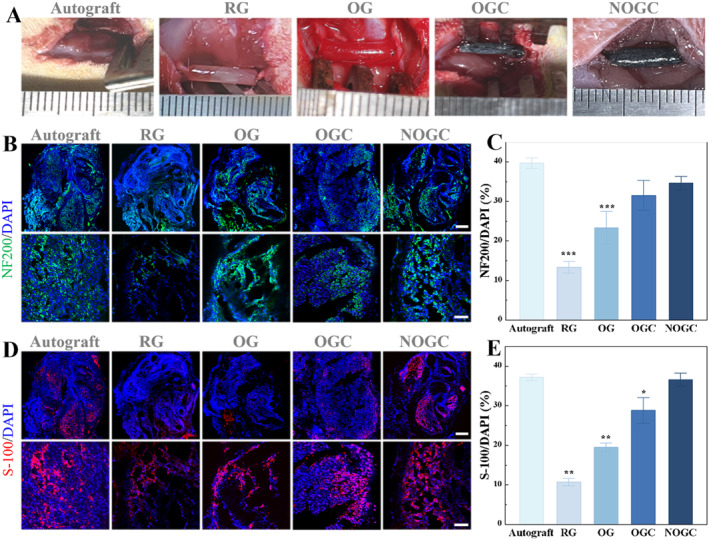
(A) Representative photographs of the rat models of sciatic nerve defects with different treatments. (B) Immunofluorescent images of NF200 in different groups. Scale bars represent 200 μm (up) and 50 μm (down). (C) NF200‐positive area determined by immunofluorescence images. (D) Immunofluorescence images of S‐100 staining in different groups. Scale bars represent 200 μm (up) and 50 μm (down). (E) S‐100‐positive area determined by immunofluorescence images immunofluorescence images. *p < 0.05; **p < 0.01; ***p < 0.001.

To evaluate the potential of conductive NGCs with orientated topological structures in promoting nerve repair and functional restoration, the prepared conduit was implanted into a 10 mm gap in a rat's sciatic nerve and treated for 8 weeks. The rats were divided into five groups, each receiving a different treatment, namely Autograft, randomly arranged GelMA (RG) conduit, OG conduit, OGC conduit, and NOGC conduit (Figure 4A). At the 8‐week postoperative interval, the NGCs remained in situ and exhibited signs of degradation, thereby revealing its biodegradability and eliminating the necessity for a subsequent surgical removal (Figure S4). Furthermore, the regenerated nerves were isolated and assessed through immunofluorescent staining (Figure 4B,D). It was found that the RG group exhibited a reduced number of Schwann cells and a sparser arrangement of neurofilaments in comparison to the OG group due to the directional induction effect of the topological structure on the regenerative nerve. Besides, the doping of conductive CNTs resulted in denser neurofilaments and more mature Schwann cells in the OGC group than that in the OG group, which was ascribed to the electrical stimulation of CNTs. Moreover, the regenerated nerves in the NOGC group were comparable to those in the Autograft group, displaying mature myelination and dense nerve filaments compared with other groups. In addition, quantitative analysis of the positive expression of NF200 and S‐100 in these groups was performed, as shown in Figure 4C,E. These results indicated that the prepared conductive nerve conduit exhibited the following advantages: a topological structure directing nerve growth, conductivity stimulating cell growth and differentiation, and released NGF promoting cell differentiation.

**FIGURE 5:**
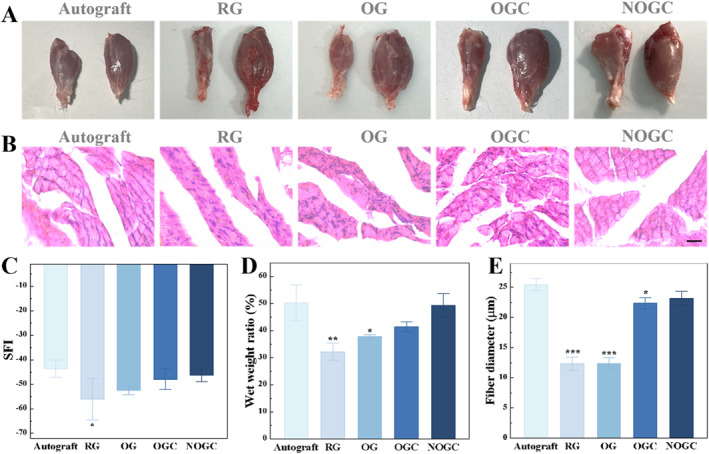
(A) Photographs of gastrocnemius muscles of rats in five groups. (B) HE staining images of rat muscle sections in five groups. Scale bar is 20 μm. (C) SFI of rats in five groups. (D) Statistics of the wet weight of isolated rat hind leg gastrocnemius in five groups. (E) Gastrocnemius muscle fiber diameter of rats in five groups. *p < 0.05; **p < 0.01; ***p < 0.001.

Additionally, to examine the motor function of the innervated muscle, walking track analysis and evaluation of the gastrocnemius muscles were conducted (Figure 5). One crucial metric for evaluating the recovery of motor function is the sciatic nerve function index (SFI). Normal function is represented by an SFI of 0, whereas a complete loss of function is represented by an SFI of −100. At 8 weeks after implantation, the NOGC group showed similar SFI to the Autograft group (Figure S5 and 5C). Besides, the gastrocnemius muscles on the surgical the non‐surgical sides were isolated, and the wet weight ratio was recorded. It was evident that the rats in the RG group showed severe muscle atrophy on the surgical side, whereas those in the Autograft and NOGC groups exhibited atrophy recovery (Figure 5A). The ratio of wet weight in the experimental and normal gastrocnemius muscle displayed an increasing trend in the RG, OG, OGC and NOGC groups (Figure 5D). Significantly, the NOGC group showed a comparable gastrocnemius wet weight ratio to the Autograft group. Moreover, the state of muscle fibers was ascertained using hematoxylin and eosin (HE) staining. As shown in Figure 5B,E, the diameters of muscle fibers in the Autograft and NOGC groups were significantly larger than those observed in the other groups. These findings stated that the NOGC scaffold demonstrated motor function recovery comparable to autograft, suggesting its potential as a viable alternative for neural tissue regeneration.

## 3 Conclusion

In summary, we developed a type of conductive NGC featuring a directional topology through ice‐templating technology for PNI repair. The temperature gradient generated by the Peltier thermoelectric cooling platform directed the growth of ice crystals, thereby imparting a directional structure to the conduit's internal wall. This unique topological feature encourages neurons to grow directionally within the confined space. Furthermore, the conduit's conductivity facilitates the transmission of electrical signals essential for neuronal growth, augmenting the efficiency of nerve reconstruction. We also incorporated NGF into the conduit to improve its bioactivity for injured nerve repair. These conductive NGCs were confirmed to effectively stimulate cell differentiation and direct neurite elongation. Further animal studies on rats with long‐segment sciatic nerve defects demonstrated that the NGCs accelerated nerve repair and functional recovery. These results underscore the clinical potential of such NGCs in improving nerve regeneration.

## 4 Experimental Section/Methods

### 4.1 Synthesis of GelMA

Gelatin (10 g) was weighed into a bottle with 100 mL PBS, heated, and stirred in a 60°C water bath until completely dissolved. Simultaneously, additional 400 mL PBS was heated to 60°C. Methacrylic anhydride (10 mL) was added dropwise, and the reaction occurred in a 60°C water bath for 2.5 h. After that, the 400 mL heated PBS was added, and the reaction continued for 1.5 h. Then, the solution was dialyzed in a 45°C water bath for 7 days, followed by freeze‐drying.

### 4.2 Preparation of Conductive Hydrogel Precursor Solution

A 10 wt% GelMA hydrogel precursor solution was meticulously prepared by combining lyophilized GelMA sponge with ddH_2_O and heating the solution at 55°C until fully dissolved. Following this, 2 vol% of a photoinitiator, specifically hydroxy‐2‐methyl‐1‐phenyl‐1‐propanone, was introduced to the solution. The resultant mixture was then thoroughly and uniformly mixed using ultrasonic waves, all within a dark environment. Subsequently, 20 mg of CNTs were introduced and homogeneously dispersed into the already mixed solution (0.5 mL), thus, the conductive hydrogel precursor solution was obtained.

### 4.3 Construction of Nerve Conduits With Topological Structure

A sandwich conduit perfusion mold was constructed using glass capillaries with varying inner diameters. The outer diameter of the inside glass capillary was 3.6 mm, while the inner diameter of the outside glass capillary was 4.4 mm. The apparatus was placed on a thermoelectric cooling platform and the conductive hydrogel precursor solution was added to the interlayer between the two capillaries, thereby producing a bottom‐up temperature gradient and a directional topology. Concurrently, to circumvent a substantial temperature discrepancy between the thermoelectric cooling platform and the surrounding environment, as well as the premature freezing of the hydrogel solution, which was susceptible to the ambient temperature and transformed into a colloidal substance, a sponge insulation was incorporated outside the double‐layer glass capillary. Once the solution within the glass capillary had reached its solidification point, UV light was applied to the conduit in order to fully irradiate it, thereby initiating the crosslinking process of the hydrogel. The resulting hollow conductive nerve conduit with a topological structure was then extracted from the glass capillaries.

### 4.4 PC12 Cell Cultivation

PC12 cells were grown in culture medium (RPMI 1640) supplemented with 10 vol% fetal bovine serum and 1 vol% penicillin‐streptomycin and maintained at 37°C under 5% CO_2_. For differentiation, 50 ng/mL NGF was added to the medium. The cells were cultivated for 5 days, with medium changes every 2 days. Moreover, PC12 cells were seeded on four different substrates: TCP, OG, OGC, and NOGC. Except for NOGC, the other groups were treated with free NGF. After 7 days of culture, cells were fixed, immunostained for neuronal markers, and imaged by confocal laser scanning microscopy to assess neurite outgrowth.

### 4.5 Immunofluorescence Staining

The cell samples were immobilized using 4% formaldehyde at room temperature for 45 min. They were made permeable by soaking in PBS mixed with 0.1% Triton X‐100 for 15 min and blocked using bovine serum albumin for 1 h. Afterward, the samples were left to incubate with the primary antibody against βIII‐tubulin overnight. The next day, the samples were rinsed three times with PBS and incubated for another hour with a mixture of DAPI and the corresponding secondary antibody. Lastly, the samples were cleaned, encapsulated into the antifade mounting medium with coverslips, and observed under a laser scanning confocal microscope.

### 4.6 Animal Experiments

Defective sciatic nerve models were created in adult male SD rats. After anesthesia in the rat, the procedure commenced with the exposure of the sciatic nerve through an incision in the skin and muscle of the left leg. The nerve was then transected, and a 10 mm segment was removed, leaving a gap. After that, 12 mm of NGCs were implanted to bridge the two ends of the defect, with the epineurium sheath securely fastened using 8‐0 nylon sutures. In the autologous transplantation group, the proximal and distal nerve ends were exchanged and sutured together after the creation of a similar gap. The muscle and skin were then stitched carefully. After 8 weeks, the rats were euthanized and examined.

### 4.7 Footprint Analysis

Following an 8‐week period of NGC implantation, footprint analysis was performed on all animals to evaluate functional recovery. The rats' hind feet were coated with carbon ink, and they were then directed to walk across a piece of paper, leaving their footprints. The length of the print, as well as the spread of the toes and middle toe, were measured on both the left and right sides of each footprint.

### 4.8 Evaluation of Regenerated Nerves

At 8 weeks, the surgical site was exposed and regenerated nerves were isolated. Following an overnight fixation with 4% paraformaldehyde solution at 4°C, the nerves were embedded and frozen in the OCT compound. The OCT‐embedded regenerated nerves were cut into sections with a thickness of 10 μm using a freezing microtome. Subsequently, the sections obtained were stained with NF200 and S‐100β in accordance with the aforementioned immunofluorescence staining procedure. Ultimately, the stained sections were examined under a laser scanning confocal microscope.

### 4.9 Statistical Analysis

For image processing and statistical analysis, Image J and Origin software were utilized. The experimental data underwent analysis using one‐way ANOVA test.

## Author Contributions

H.Z. and K.C.W. contributed equally to this work. H.W. conceived the idea. K.C.W. conducted experiments. K.C.W. and H.Z. carried out data analysis and wrote the manuscript. H.Z., D.Y.X., S.S.M., and H.W. revised the manuscript. S.S.M., Y.H.D., P.M.L., and H.W. contributed to the scientific discussion of the article.

## Ethics Statement

The Animal Ethics Committee of Zhongda Hospital, Southeast University School of Medicine, approved all animal experimental procedures (20230625001).

## Conflicts of Interest

The authors declare no conflicts of interest.

## Supporting information

Figures S1–S5

## Data Availability

The data that support the findings of this study are available from the corresponding author upon reasonable request.
