# Supplementary material for: Conductive Nerve Conduits With Orientated Topological Structures From Ice‐Templating Technology
Source: Smart Med. 2025 Jun 30;4(3):e70012. doi: 10.1002/smmd.70012 (PMC12224919; doi:10.1002/smmd.70012)
Supplement: Supplementary file 1 — Figures S1–S5 [file SMMD-4-e70012-s001.docx]

Supporting Information

Conductive nerve conduits with orientated topological structures from ice-templating technology

Hui Zhang, Kaichen Wang, Dongyu Xu, Shuangshuang Miao, Yanhong Dai^*^, Panmiao Liu^*^, Huan Wang^*^


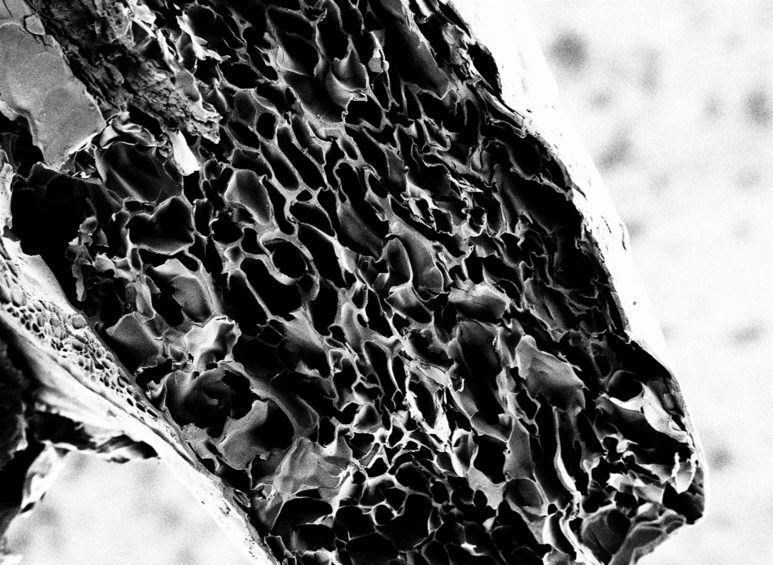


**Figure S1.** SEM image of the cross-section of the conduit.


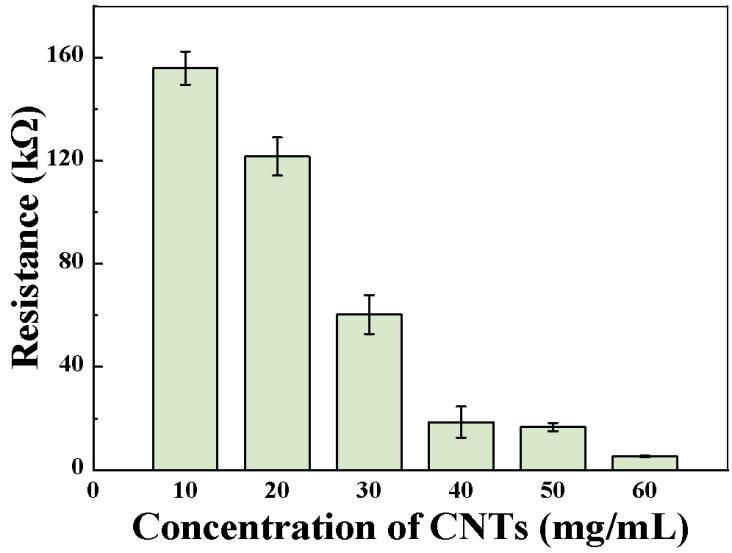


**Figure S2.** Resistance of hydrogels with different concentrations of CNTs.


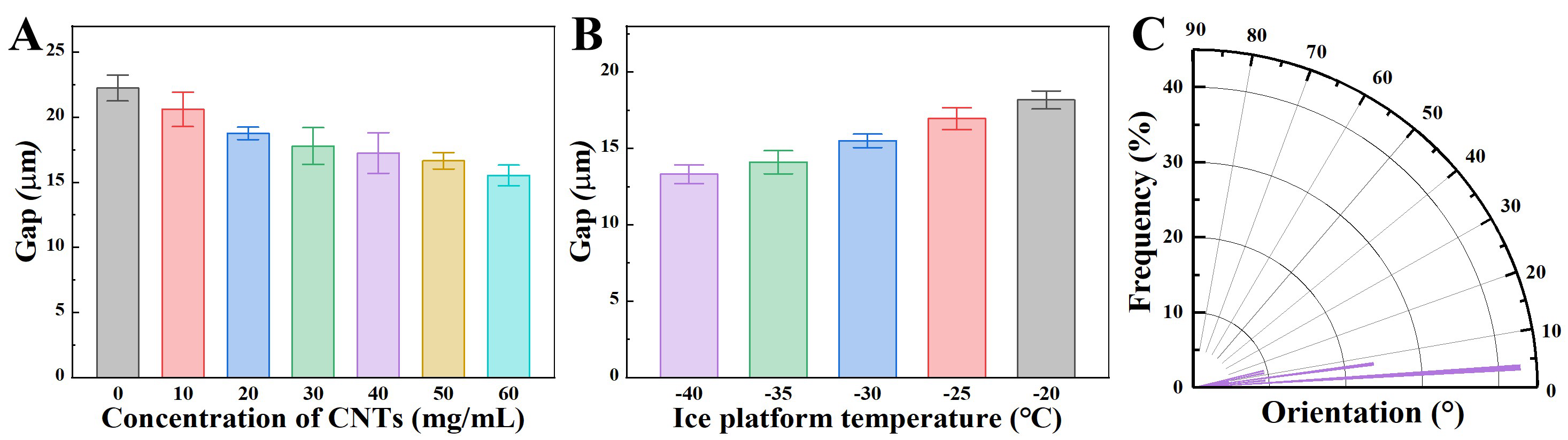


**Figure S3.** (A) Gap size of hydrogels incorporating various concentrations of CNTs. (B) Gap size of hydrogels prepared under different temperatures of ice platform. (C) Orientation analysis of gaps.


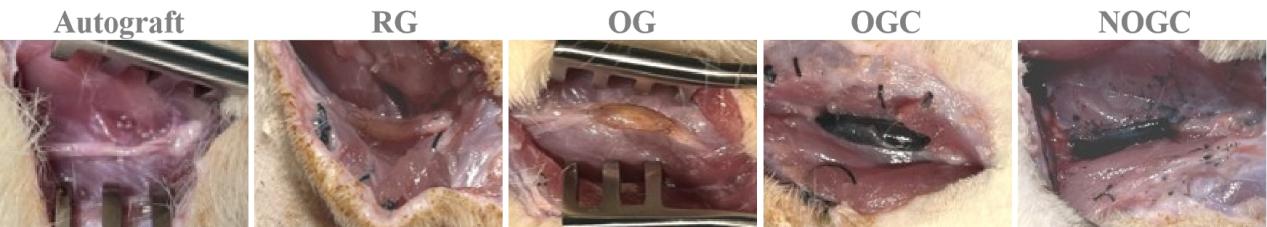


**Figure S****4.** Regenerated nerves of rats with different treatments at 8 weeks post-operation.


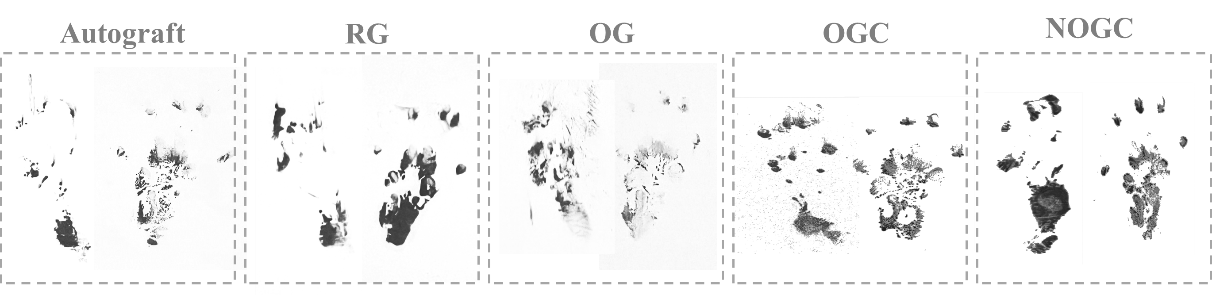


**Figure S5.** Representative pictures of rat footprints with different treatments after 8 weeks.
